# Lesinurad, a novel, oral compound for gout, acts to decrease serum uric acid through inhibition of urate transporters in the kidney

**DOI:** 10.1186/s13075-016-1107-x

**Published:** 2016-10-03

**Authors:** Jeffrey Miner, Philip K. Tan, David Hyndman, Sha Liu, Cory Iverson, Payal Nanavati, David T. Hagerty, Kimberly Manhard, Zancong Shen, Jean-Luc Girardet, Li-Tain Yeh, Robert Terkeltaub, Barry Quart

**Affiliations:** 1 Ardea Biosciences, Inc., 9390 Towne Centre Drive, San Diego, CA 92121 USA; 2 University of California San Diego, 9500 Gilman Dr, La Jolla, CA 92093 USA

**Keywords:** Gout, RDEA594, Uric Acid, URAT1

## Abstract

**Background:**

Excess body burden of uric acid promotes gout. Diminished renal clearance of uric acid causes hyperuricemia in most patients with gout, and the renal urate transporter (URAT)1 is important for regulation of serum uric acid (sUA) levels. The URAT1 inhibitors probenecid and benzbromarone are used as gout therapies; however, their use is limited by drug–drug interactions and off-target toxicity, respectively. Here, we define the mechanism of action of lesinurad (Zurampic®; RDEA594), a novel URAT1 inhibitor, recently approved in the USA and Europe for treatment of chronic gout.

**Methods:**

sUA levels, fractional excretion of uric acid (FE_UA_), lesinurad plasma levels, and urinary excretion of lesinurad were measured in healthy volunteers treated with lesinurad. In addition, lesinurad, probenecid, and benzbromarone were compared in vitro for effects on urate transporters and the organic anion transporters (OAT)1 and OAT3, changes in mitochondrial membrane potential, and human peroxisome proliferator-activated receptor gamma (PPARγ) activity.

**Results:**

After 6 hours, a single 200-mg dose of lesinurad elevated FE_UA_ 3.6-fold (*p* < 0.001) and reduced sUA levels by 33 % (*p* < 0.001). At concentrations achieved in the clinic, lesinurad inhibited activity of URAT1 and OAT4 in vitro, did not inhibit GLUT9, and had no effect on ABCG2. Lesinurad also showed a low risk for mitochondrial toxicity and PPARγ induction compared to benzbromarone. Unlike probenecid, lesinurad did not inhibit OAT1 or OAT3 in the clinical setting.

**Conclusion:**

The pharmacodynamic effects and in vitro activity of lesinurad are consistent with inhibition of URAT1 and OAT4, major apical transporters for uric acid. Lesinurad also has a favorable selectivity and safety profile, consistent with an important role in sUA-lowering therapy for patients with gout.

**Electronic supplementary material:**

The online version of this article (doi:10.1186/s13075-016-1107-x) contains supplementary material, which is available to authorized users.

## Background

Gout is a urate crystal deposition disease that results from a metabolic disorder, hyperuricemia (elevated serum uric acid (sUA)), and the consequent deposition of monosodium urate crystals in joints, soft tissues, and organs [1]. Gout is a major health problem and is increasing in prevalence in the USA and many other countries [2–7]. Primary prevention and treatment of gout includes measures to keep sUA levels below 6 mg/dL [8–12]. Diminished renal clearance of uric acid promotes hyperuricemia in at least 90 % of patients with gout [13]. Thus treatment with an agent that has a uric acid lowering effect by increasing urinary excretion could be useful in these patients.

Renal factors contributing to hyperuricemia include single or combined effects of decreased glomerular filtration rate, drug-induced increases in renal urate reabsorption, and altered expression and/or function of renal urate transporters [14]. Serum urate is normally filtered by the glomerulus, with near complete reabsorption in the kidney proximal tubule [15]. Genome-wide association studies show that multiple renal urate transporters contribute to the regulation of sUA levels [16–18], and genetic loss of function studies indicate that URAT1 (*SLC22A12*) and GLUT9 (*SLC2A9*) play significant roles in renal reabsorption of urate, as patients with disruptive mutations in these transporters have high fractional excretion of uric acid (FE_UA_) and hypo-uricemia [19–23]. Organic anion transporter (OAT)4, is another urate transporter that has been genetically associated with hyperuricemia and gout [18, 24], and with an increased risk of hyperuricemia and gout induced by treatment with diuretics [25–28].

Management approaches to hyperuricemia in gout include not only xanthine oxidase inhibitor (XOI) monotherapy and uricase therapy for refractory disease, but also URAT1 inhibitors (probenecid and benzbromarone) in monotherapy or in combination with XO inhibition [9]. However, probenecid is limited by the need to dose two to four times daily due to its short half-life. Probenecid also alters the pharmacokinetics (PK) of a wide variety of other drugs through inhibition of OAT1 and OAT3 (OAT1/*SLC22A6* and OAT3/*SLC22A8*) [29, 30]. These drug–drug interactions limit probenecid use and increase the complexity of dosing in combination with other therapies. Benzbromarone is a highly potent URAT1 inhibitor that is not available in the USA and has very limited availability in Europe, because of links with idiosyncratic, but severe hepatotoxicity [31]. Other drugs with mild-to-moderate URAT1 inhibition activity, such as the lipid-lowering agent, fenofibrate, and the angiotensin receptor blocker, losartan, have been studied for the treatment of hyperuricemia and gout [32, 33], but there remains an important clinical need for potent and selective URAT1 inhibitors. In patients with gout, restoring normal uric acid excretion with a URAT1 inhibitor in combination with an XOI represents a potentially powerful approach to lowering sUA [34].

**Fig. 1:**
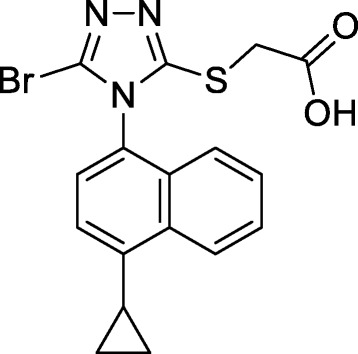
Structure of lesinurad (RDEA594), 2-((5-bromo-4-(4-cyclopropylnaphthalen-1-yl)-4*H*-1,2,4-triazol-3-yl)thio) acetic acid

Lesinurad (Fig. 1), a metabolite of another compound in development, was discovered to be responsible for an unexpected decrease in sUA levels seen in early clinical trials. Lesinurad exhibited significant urinary excretion that was closely correlated with urinary uric acid excretion [35]. These findings suggest that the decreased sUA was occurring through effects on renal urate handling caused by lesinurad. A potential target is URAT1, a transporter known to be associated with uric acid reabsorption [19]; however, other transporters are also known to be involved [36]. Lesinurad reduced sUA and increased fractional excretion of uric acid (FE_UA_) in a dose-dependent manner in healthy volunteers after a single dose, consistent with the inhibition of URAT1 and OAT4 observed in vitro. Importantly no clinically relevant changes in the PK of lesinurad or the XOIs febuxostat or allopurinol have been detected in patients with gout [34, 37].

In this study, we define the molecular mechanism of action of the uric acid reabsorption inhibitor lesinurad (Zurampic®), previously known as RDEA594. Lesinurad is an oral agent recently approved in its 200-mg dose by the US Food and Drug Administration [38] and the European Commission. It is indicated in combination with an XOI for the treatment of hyperuricemia associated with gout in patients who have not achieved target sUA levels with an XOI alone.

## Methods

### Transporter activity assays

For URAT1 and OAT4, DNA constructs pCMV/neo-URAT1 and pCMV/neo-OAT4 were purchased from Origene Technologies. In addition, a glycine codon was introduced just prior to the stop codon using the QuikChange Site-Directed Mutagenesis Kit (Agilent Technologies) according to the manufacturer’s instructions, producing pCMV/neo-URAT1-553G and pCMV/neo-OAT4-551G. The added glycine has no effect on the activity of the proteins (data not shown). Stably transfected HEK293-OAT4-551G cells were used for OAT4 transport activity assays, which were grown for several weeks in media (DMEM with 10 % fetal bovine serum and 1 mM sodium pyruvate) containing 500 μg/mL geneticin sulfate. An isolated clone with high OAT4 activity was selected for activity assays. One day prior to the assay, cells were plated at 200,000 cells/well into each well of white, clear bottom poly-D-lysine-coated assay plates (BD Biosciences). URAT1-553G-expressing cells were prepared by transient reverse transfection of pCMV/neo-URAT1-553G into HEK-293 T cells using DreamFect Gold (Boca Scientific) according to the manufacturer’s instructions, and plated directly into assay plates in the same way as the OAT4-551G cells.

The cells were assayed on the following day. For measuring urate transport, cells were washed once with wash buffer (25 mM MES pH 5.5, 125 mM sodium gluconate), and then incubated in assay buffer (25 mM MES pH 5.5, 125 mM sodium gluconate, 4.8 mM potassium gluconate, 1.2 mM KH_2_PO_4_, 1.2 mM MgSO_4_, 1.3 mM calcium gluconate, and 5.6 mM glucose) containing different amounts of lesinurad, for 5 minutes: ^14^C-urate (American Radiolabeled Chemicals, Inc.) at a final concentration of 100 μM in assay buffer was then added and incubated with the cells for 10 minutes. The free urate was removed by aspiration and the cells rinsed three times in 150 μL wash buffer. The cells were then subjected to liquid scintillation counting to measure transported urate. Lesinurad dose-response curves and half-maximal inhibition constants (IC_50_ values) were generated from the urate transport results using GraphPad Prism software (GraphPad Software, Inc.), variable slope (four parameter) model. Each point represents the mean and standard error of the mean (SEM) from triplicates.

For OAT1, HEK-293 T cells were transfected as described above using pSPORT6-hOAT1 (Thermo Scientific) and assayed for transport of 5 μM 6-carboxyfluorescein (CF) for 2 minutes with or without lesinurad, in assay buffer containing 125 mM NaCl, 4.8 mM KCl, 5.6 mM D-glucose, 1.2 mM CaCl_2_, 1.2 mM KH_2_PO_4_, 1.2 mM MgSO_4_, and 25 mM HEPES, pH 7.3. The cells were washed three times in a buffer containing 125 mM NaCl and 25 mM HEPES, pH 7.3, and then lysed in 1 M sodium hydroxide prior to fluorescence measurement.

For ABCG2, monolayers of Caco-2 cells expressing ABCG2 were grown on a permeable support (1 μM PET membranes) in 24-transwell plates. Cells were washed and incubated in Hank’s balanced salt solution, with or without different concentrations of lesinurad or with 100 μM chrysin for 30 minutes at 37 °C in both apical (A) and basolateral (B) compartments. Each treatment was assayed in triplicate: [^3^H]-genistein substrate at 2 nM was then added for an additional 120 minutes at 37 °C. For basolateral-to-apical (B → A) flux measurements, genistein was placed in the basolateral compartment only (donor well) and a sample was taken after the 120-minute incubation period from the apical compartment (receiver well). For apical-to-basolateral (A → B) flux measurements, genistein was placed in the apical compartment only (donor well) and a sample was taken after the 120-minute incubation period from the basolateral compartment (receiver well). Samples were then subjected to scintillation counting. The inhibition of BCRP/ABCG2 was then calculated by the net B → A flux method [39]. The substrate flux (A → B or B → A) is determined by the apparent permeability (P_app_) using the following equation:

$$ {\mathrm{P}}_{\mathrm{app}} = \left[1/\left(\mathrm{Area}\ *\ {\mathrm{C}}_{\mathrm{D}}(0)\right)\right]\ *\ {\mathrm{dM}}_{\mathrm{r}}/\mathrm{d}\mathrm{t}. $$

where Area is the area of the filter in the transwell, C_D_(0) is the initial dosing concentration of genistein, and dM_r_/dt is the slope of the flux divided by the incubation time.

The net transport in the B → A direction was determined by subtracting the substrate flux in the A → B direction from that in the B → A direction:

$$ \mathrm{N}\mathrm{e}\mathrm{t}\ \mathrm{B}\ \to\ \mathrm{A}\ \mathrm{flux} = {\mathrm{P}}_{\mathrm{app}}\left(\mathrm{B}\ \to\ \mathrm{A}\right)\hbox{-} {\mathrm{P}}_{\mathrm{app}}\left(\mathrm{A}\to \mathrm{B}\right). $$

Percent inhibition is calculated by dividing the Net (B → A) flux in the presence of the inhibitor by the Net (B → A) flux in the absence of the inhibitor (inh):

$$ \mathrm{Percent}\ \mathrm{Inhibition} = 100\ \hbox{-} \left[100*\mathrm{N}\mathrm{e}\mathrm{t}{\left(\mathrm{B}\to \mathrm{A}\right)}_{\mathrm{inh}}/\mathrm{mean}\ \mathrm{N}\mathrm{e}\mathrm{t}\left(\mathrm{B}\to \mathrm{A}\right)\right]. $$

For GLUT9, SLC2A9v2/GLUT9ΔN in pCMV6/neo was linearized with *Xma*I and *Not*I and cRNA transcribed using the mMessage mMachine T7 kit (Ambion). Oocytes were injected with either water or 25 ng cRNA and incubated for 3 to 5 days in ND96 buffer. For each point, 10 oocytes were incubated with 100 μM ^14^C-uric acid in ND96 buffer with and without test compounds at room temperature for 60 minutes. The oocytes were then washed three to four times in ice-cold ND96 buffer and lysed prior to scintillation counting.

### Mitochondrial toxicity assay

HepG2 cells were counted via hemocytometer and diluted into equal volumes of serum-free Opti-MEM (Life Technologies) and JC-1 Staining Solution (Sigma catalog number CS0390) at 300,000 cells/mL in the presence of 2.5 μg/mL JC-1. Cells were incubated at 37 °C and 5 % CO2 for 20 minutes, rinsed once with Hank's Balanced Salt Solution, and resuspended to the same concentration in serum-free Opti-MEM. Cells were then seeded into 96-well black-sided clear-bottom tissue culture plates at 30,000 cells/100 μL/well. Three replicates per experimental condition group were used. Compound was added with the final dimethyl sulfoxide (DMSO) concentration at 0.5 %. After 2 hours at 37 °C and 5 % CO2, medium was aspirated and plates were read on a Gemini EM spectrofluorometer (Molecular Devices). Fluorescence of JC-1 monomers was monitored by excitation at 490 nm and emission at 530 nm (green) and of aggregates by excitation at 525 nm and emission at 590 nm (red). The ratio of J-aggregates to monomers was calculated, with a lower ratio indicating increased cell toxicity. The percent change in mitochondrial membrane potential (Ψm) was calculated as follows:

[Em590/Em530 (vehicle only) – Em590/Em530 (test compound)]/[Em590/Em530 (vehicle only) – Em590/Em530 (100 μM menadione)].

### Human peroxisome proliferator-activated receptor gamma assay

This assay was performed using nonhuman mammalian cells engineered to provide a PPARγ-responsive luciferase reporter gene and constitutive, high levels of PPARγ (Indigo Biosciences catalog number IB00101). Cells were incubated with compounds for 24 hours, and then luciferase activity was measured using a luminometer. Agonist activity was reported as fold activation over a vehicle control. A positive control agonist, rosiglitazone, was included in each batch run to verify cell integrity.

### Clinical studies

#### Lesinurad effects on FE_UA_

From a phase 1 clinical trial, RDEA594-109, eight healthy male volunteers were administered single oral doses of 200, 400, or 600 mg lesinurad tablets (with food). In each treatment period, plasma samples were collected within 30 minutes before dosing (pre-dose) and at 0.5, 1, 1.5, 2, 3, 4, 5, 6, 8, 10, 12, 16, 24, 30, 36, and 48 hours post-dose. Urine (total catch) was collected pretreatment at hours –24 to –18, –18 to –12, and –12 to 0, and post-dose at hours 0 to 6, 6 to 12, 12 to 24, 24 to 30, 30 to 36, and 36 to 48.

After protein precipitation of the samples, lesinurad levels in plasma and urine were measured at Ardea Biosciences by quantitative high performance liquid chromatography with tandem mass spectrometry. The lesinurad plasma PK parameters maximum plasma concentration (C_max_) and area under the curve (AUC) were derived using the software WinNonlin Professional, Version 5.2 (Pharsight Corporation). The parameters from individual profiles of lesinurad were determined using noncompartmental methods. Urine samples were analyzed for uric acid and creatinine using enzymatic methods by Covance Clinical Research Unit (Dallas, TX, USA), and pharmacodynamic (PD) parameters were determined by Covance Clinical Research Unit using SAS, Version 8.2 (SAS Institute Inc). Calculated PD parameters for uric acid included urinary excretion, renal clearance, and FE_UA_. FE_UA_ was calculated by dividing the uric acid clearance by the creatinine clearance. For analyzing the relationship between plasma lesinurad concentrations and FE_UA_, the weighted average plasma lesinurad concentration was calculated for plasma samples from each time period that corresponded to the associated urine samples.

#### Lesinurad effects on sUA

Serum was collected within 30 minutes prior to drug administration, and at 6, 12, 16, 24, 30, 36, and 48 hours after dosing. Serum urate levels and PD parameters were assayed and calculated by Covance Clinical Research Unit as described above.

## Results

### Clinical effects on urate handling and pharmacokinetics of lesinurad

**Fig. 2:**
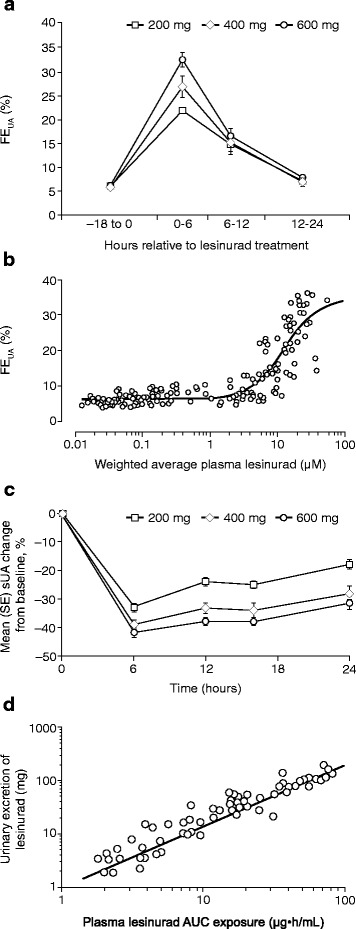
Effects of lesinurad on urate handling and lesinurad pharmacokinetics in healthy human volunteers. **a** Dose-dependent increase in fractional excretion of uric acid (*FE*_*UA*_) after a single 200-mg, 400-mg, or 600-mg dose of lesinurad. FE_UA_ was elevated from baseline (–18 to 0 hours) at 0–6 hours after dosing and then returned to normal after 24 hours. For all doses, *p* < 0.001 at 0–6 hours and 6–12 hours relative to baseline. **b** FE_UA_ levels increased with increasing plasma lesinurad. The half-maximal effective concentration of plasma lesinurad concentrations on FE_UA_ was 13 μM. Results are from healthy volunteers on a single 200-mg, 400-mg, or 600-mg dose of lesinurad. **c** Healthy volunteers on a single 200-mg, 400-mg, or 600-mg dose of lesinurad had a dose-dependent decrease in serum uric acid (*sUA*), for every drug treatment point for all doses relative to baseline, *p* <0.001. **d** Urinary excretion of lesinurad correlated linearly with plasma lesinurad following a single dose of 200, 400, or 600 mg lesinurad. *AUC* area under the curve for plasma concentration vs. time

To determine the effects of lesinurad on urate handling, the levels of uric acid and lesinurad were measured in the blood and urine in healthy volunteers (Fig. 2). Lesinurad produced a dose-dependent increase in FE_UA_, a measure of the relative amount of uric acid excreted by the kidneys (Fig. 2a). After a single 200-mg, 400-mg, and 600-mg dose of lesinurad, the FE_UA_ increased from a baseline of 5.8 % to a peak of 21.8, 26.7, and 32.3 %, respectively, within 6 hours, and then dropped to baseline levels by 24 hours. FE_UA_ increased with lesinurad plasma concentrations (Fig. 2b). The half-maximal effective plasma concentration of lesinurad for elevating FE_UA_ was 13 μM, and some individuals with high plasma lesinurad had up to 35 % FE_UA_. In accord with the increases in FE_UA_, single doses of lesinurad led to a dose-dependent reduction in sUA (Fig. 2c). The highest 600-mg dose of lesinurad reduced sUA by a maximum of 42 % after 6 hours, and this was sustained at 31 % after 24 hours. The lower 200-mg and 400-mg dose of lesinurad also reduced sUA dramatically, albeit less so compared with the 600-mg dose. Therefore, lesinurad increased the renal excretion of uric acid and lowered sUA. In addition, lesinurad underwent active urinary excretion, which correlated linearly with the plasma concentration of lesinurad (Fig. 2d).

### Inhibition of reabsorptive urate transporters by lesinurad

**Fig. 3:**
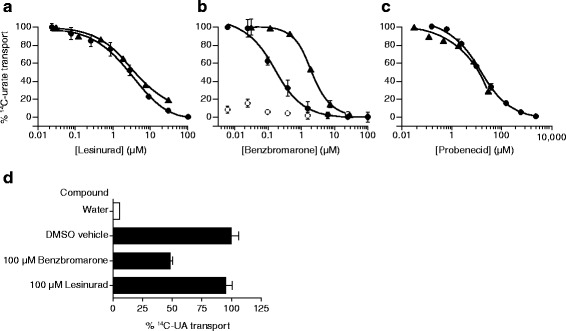
Lesinurad inhibits URAT1 and OAT4 urate-transport activity with equal potency, and does not inhibit GLUT9. Urate transport was measured in cells expressing URAT1-553G (*closed circles*) or OAT4-551G (*triangles*) treated with different amounts of lesinurad (**a**) (*left panel*), benzbromarone (**b**) (*center panel*), or probenecid (**c**) (*right panel*). Untransfected cells (**b**) (*open circles*) were used to reveal the background uric acid transport in the cells. Results are from a single experiment and are representative of multiple experiments. Each point is from samples performed in triplicate, presented as mean ± SEM. **d** Lesinurad does not inhibit GLUT9. Urate transport was measured in oocytes injected with water and treated with dimethyl sulfoxide (*DMSO*) (*open bar*), or in oocytes injected with cRNA expressing GLUT9 (SLC2a9v2/GLUT9ΔN) (*solid bars*) and either treated with DMSO, 100 μM benzbromarone, or 100 μM lesinurad. Benzbromarone inhibited GLUT9, as shown previously [21, 55]. Results are shown in mean ± SEM. SLC2A9v1/GLUT9 was also examined, yielding similar results. *UA* uric acid

The urate transporters URAT1, OAT4, and GLUT9 are all genetically associated with sUA and promote the reabsorption of uric acid from the kidney proximal tubule. The urate-lowering gout therapies benzbromarone and probenecid block renal reabsorption of uric acid and are known URAT1 inhibitors. We measured the potency of lesinurad, benzbromarone, and probenecid against URAT1 and OAT4 (Table 1 and Fig. 3). Lesinurad inhibited the urate-transport activity of URAT1 and OAT4 in a dose-dependent manner and at a similar potency, with an IC_50_ of 3.53 and 2.03 μM, respectively (Fig. 3a). Benzbromarone inhibited URAT1 and OAT4 with an IC_50_ of 0.29 and 3.19 μM, respectively (Fig. 3b). Probenecid inhibited both transporters equipotently at 13.23 (URAT1) and 15.54 (OAT4) μM, but with lower potency than lesinurad (Fig. 3c). Lesinurad at up to 100 μM had no effect on GLUT9 (Fig. 3d). Benzbromarone had only weak activity on GLUT9 and is not likely to be active in vivo on this transporter.

**Table 1:** Potencies (μM, mean ± SEM) of lesinurad, benzbromarone, and probenecid against the resorptive uric acid transporters URAT1, OAT4, and GLUT9

	Reabsorptive uric acid transporter		
Compound	URAT1	OAT4	GLUT9
Lesinurad	3.53 ± 0.52	2.03 ± 0.66	>100
Benzbromarone	0.29 ± 0.06	3.19 ± 1.04	~100
Probenecid	13.23 ± 0.44	15.54 ± 3.39	ND

Results are from three experimentsfree concentration

### Transporters involved in urate secretion and drug–drug interactions

**Fig. 4:**
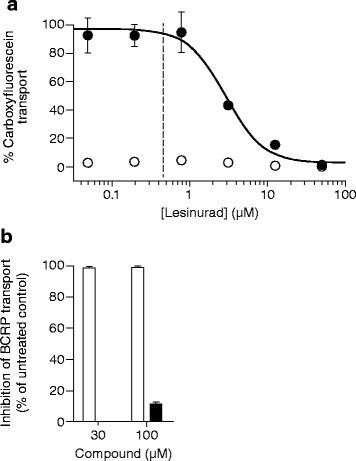
Effects of lesinurad on OAT1 and ABCG2. **a** Lesinurad inhibits OAT1. Cells transfected with control plasmid (pSPORT6, *open circles*) or OAT1-expressing plasmid (pSPORT6-hOAT1) were incubated with different concentrations of lesinurad. Data are from an individual representative experiment, shown as the mean ± SEM of samples in triplicate. The *dashed line* indicates the maximum plasma- free concentration (C_max,free_) of a single 200-mg dose of lesinurad obtained in the clinic. This concentration does not inhibit OAT1 in vitro. **b** Lesinurad does not affect ABCG2 activity. Caco-2 cells were either untreated or treated with the indicated concentrations of lesinurad (*open bars*) for 30 minutes prior to the addition of the ABCG2 substrate genistein for 120 minutes. The inhibitor chrysin was used as a positive control (*solid bar*). Results are the mean ± SEM from samples in triplicate

ABCG2, OAT1, and OAT3 are important in the renal secretion of many drugs, endogenous organic compounds including uric acid, xenobiotics, and toxins [30, 40]. Therefore, we measured the potency of lesinurad against these transporters (Table 2 and Fig. 4). Lesinurad inhibited OAT1 with an IC_50_ of 3.90 μM (Fig. 4a) and OAT3 with an IC_50_ of 3.54 μM (previously reported in Shen et al. [41], and shown in Additional file 1: Figure S1). Lesinurad at up to 100 μM had no effect on ABCG2 (Fig. 4b).

**Table 2:** Potency of lesinurad (μM, mean ± SEM) against the secretory uric acid transporters OAT1 and ABCG2

	Secretory uric acid transporter	
Compound	OAT1	ABCG2
Lesinurad	3.90 ± 0.94	>100

Although lesinurad inhibits URAT1, OAT1, and OAT3 at a similar potency, lesinurad does not inhibit OAT1 or OAT3 in vivo [41]. For a single 200-mg dose of lesinurad, the maximal plasma concentration is 29 μM and due to extensive 98.4 % plasma protein binding [41], the predicted maximal plasma-free concentration is only 0.46 μM. This concentration is insufficient to inhibit OAT1 (Fig. 4a, dashed line).

### Mitochondrial toxicity

**Fig. 5:**
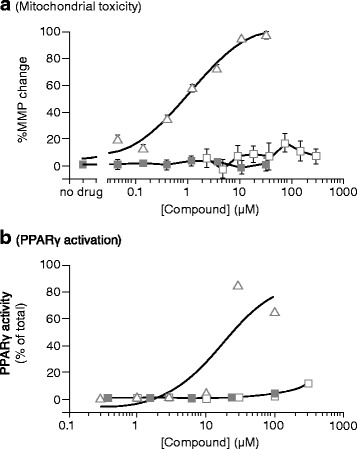
Lesinurad is not toxic to mitochondria (**a**) and does not induce peroxisome proliferator-activated receptor gamma (*PPARγ*) (**b**). Different doses of lesinurad (*closed squares*), benzbromarone (*open triangles*), or probenecid (*open squares*) were incubated with cells. **a** Mitochondrial membrane potential was measured in HepG2 cells. Lesinurad and probenecid did not change the membrane potential at any of the doses tested, whereas benzbromarone at the same concentrations altered the membrane potential in a dose-dependent manner, indicative of mitochondrial toxicity. Each point was performed in triplicate and is presented as mean ± SEM. **b** PPARγ activation was measured in cells expressing a PPARγ-responsive reporter gene. Lesinurad has no effect, although probenecid mildly induces PPARγ at the highest concentration tested. Benzbromarone strongly induces PPARγ activity. The effects of benzbromarone on perturbing hepatic mitochondrial function and activating PPARγ may lead to hepatotoxicity and cardiovascular events, respectively

Benzbromarone was never approved in the USA and was withdrawn from the European market after reports of drug-associated hepatotoxicity. Subsequently, it was demonstrated that it affects multiple mitochondrial functions that lead to mitochondrial toxicity in the human liver carcinoma cell line HepG2 and to apoptosis of rat hepatocytes [42]. Lesinurad was tested for mitochondrial toxicity with benzbromarone and probenecid in HepG2 cells (Fig. 5a). Consistent with its reported hepatotoxicity, benzbromarone reduced the mitochondrial membrane potential, a measure of mitochondrial toxicity, at concentrations as low as 0.4 μM and with a half maximal effective concentration of 1.36 μM. Although these levels are below the estimated plasma free levels, it is possible that benzbromarone and its main metabolite benzarone are concentrated to toxic levels in hepatocytes, as they are excreted primarily through the bile [43]. In contrast, lesinurad showed no mitochondrial toxicity even at the highest concentration (33 μM) tested. Probenecid showed no mitochondrial toxicity up to 300 μM.

### Peroxisome proliferator-activated receptor gamma (PPARγ) activation

PPARγ activation is associated with increased cardiovascular risk [44] and benzbromarone is known to activate PPARγ [45]. Therefore, lesinurad was tested for PPARγ activation in a cell-based reporter assay and showed no PPARγ agonism up to 100 μM (Fig. 5b), well above the C_max_ of lesinurad (Table 1). In contrast, benzbromarone strongly activated PPARγ at 30 μM, similar to previous results [45]. Probenecid showed a slight PPARγ activation at 300 μM but no activation at 100 μM or lower.

## Discussion

Lesinurad is a URAT1 inhibitor approved for the treatment of hyperuricemia associated with gout in combination with an XOI. This compound is an orally available small molecule with a structure unrelated to other known uricosuric agents. Clinical trials in healthy volunteers indicate that a single dose of lesinurad increases FE_UA_ in an exposure-dependent manner and that lesinurad treatment significantly reduces sUA (Fig. 2) [34, 35, 37]. Lesinurad reaches effective concentrations in the kidney due to active renal excretion and urinary concentration (Fig. 2d) consistent with the report that approximately one third of the compound is excreted via urine [35]. This information prompted the current investigation, in which the effects of lesinurad on the key genetically validated uric acid reabsorption transporters, URAT1, GLUT9, and OAT4 [18–24], were evaluated, demonstrating significant inhibitory activity of lesinurad on both OAT4 and URAT1, but not GLUT9.

The clinical effect of increasing fractional excretion of uric acid by lesinurad is consistent with inhibition of OAT4 and URAT1. Lesinurad-mediated inhibition of these transporters is consistent with increasing urinary uric acid excretion, and its OAT4 activity in particular may counteract diuretic-induced hyperuricemia [46, 47]. Clinical trials confirm that lesinurad adequately reduces sUA in the presence of co-administered diuretics [46, 47]. URAT1 is also the primary target of the other antihyperuricemic agents, benzbromarone and probenecid [19]. Lesinurad has a higher potency for URAT1 compared with probenecid (Fig. 3). The pharmacologic effect of benzbromarone as a URAT1 inhibitor is consistent with its estimated plasma concentration of 0.016–0.16 μM [48, 49]. Thus, benzbromarone is unlikely to inhibit GLUT9 and OAT4 due to its lower potency against these transporters (Table 1, Fig. 3b and d), low plasma concentration, and low (6 %) urinary excretion [43].

**Fig. 6:**
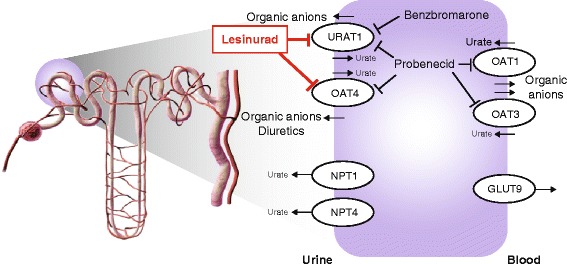
Lesinurad blocks URAT1 and OAT4 to enhance fractional excretion of uric acid and reduce serum urate levels. This diagram of a nephron depicts the location of urate transporters within the proximal tubule epithelial cell, and the mechanism of action of lesinurad compared to benzbromarone and probenecid. Lesinurad inhibits urate reabsorptive importers URAT1 and OAT4, and the inhibition of OAT4 may counteract postulated OAT4-dependent diuretic-induced hyperuricemia. Benzbromarone specifically inhibits URAT1 but not OAT4. Probenecid nonspecifically inhibits URAT1, OAT4, and other OAT family members, leading to drug–drug interactions involving OAT1 and OAT3. At physiologically relevant concentrations, none of these compounds inhibits GLUT9, another transporter that is important for the renal reabsorption of urate [18–21]

In addition to uric acid secretion, OAT1 and OAT3 are also involved in many drug–drug interactions, and hence the US Food and Drug Administration requires evaluation of the effects of new drug candidates on the activity of these transporters [50]. Despite the inhibitory activity of lesinurad on OAT1 and OAT3 in vitro, there is no effect of lesinurad on the renal clearance and PD of the OAT substrate furosemide, showing that lesinurad does not inhibit these secretory drug transporters in vivo [41]. This is in stark contrast to probenecid, which alters the PK and renal excretion of furosemide through OAT1 and OAT3 [51, 52]. Probenecid is an inhibitor of OAT1 and OAT3, and its use is limited in part due to clinically significant OAT1/OAT3-dependent drug–drug interactions [30, 53]. Plasma protein binding of lesinurad at 98.4 % [41] leads to maximal plasma-free concentrations that are insufficient to inhibit OAT1 (Fig. 4a), likely accounting for this important clinical difference between probenecid and lesinurad. Therefore, the in vivo profile of lesinurad in the human renal tubule (Fig. 6) shows inhibition of both URAT1 and OAT4 (unlike benzbromarone, which inhibits only URAT1) and no inhibition of OAT1 and OAT3 (unlike probenecid, which inhibits URAT1, OAT4, OAT1, and OAT3).

In addition to OAT1 and OAT3, the renal proximal tubule expresses a number of additional urate transporters that potentially participate in urate secretion (Fig. 6) [18, 54]. Activation of this secretory pathway could theoretically promote the observed pharmacodynamic effects of lesinurad (elevated FE_UA_ and lowered sUA). Lesinurad does not activate either OAT1 or ABCG2 (Fig. 4), two participants in the secretion of uric acid. The impact of lesinurad on other transporter candidates involved in urate secretion remains untested.

Testing benzbromarone-related endpoints, we found that lesinurad and probenecid have no effect on mitochondrial activity, in contrast to benzbromarone, which severely disrupts mitochondrial integrity in human hepatocytes at and above 0.4 μM. Similarly, with respect to PPARγ activity - and in contrast with benzbromarone - lesinurad and probenecid demonstrated no effect at up to 100 μM, thus, likely having no clinical effect based on the C_max_ of lesinurad. Benzbromarone is a known PPARγ agonist, and although no long-term cardiovascular studies have been conducted with benzbromarone, activity on this nuclear receptor is associated with increased adverse cardiovascular events [44].

## Conclusions

The action of lesinurad counteracts the inadequate excretion of uric acid commonly found in patients with gout. The potential to combine two mechanisms of treatment, inhibition of uric acid production with XOIs and inhibition of uric acid reabsorption with a selective uric acid reabsorption inhibitor, represents a powerful approach to the reduction of sUA. Lesinurad had significant URAT1 and OAT4 inhibitory activity, likely accounting for its PD effect, as well as a favorable selectivity profile in vitro compared with other drugs that inhibit uric acid reabsorption. The compound is a new treatment option for hyperuricemia associated with gout in combination with XOIs.

## Additional file

Additional file 1: Inhibition of OAT3 by lesinurad. (DOCX 59 kb)

## Abbreviations

AUC: area under the curve
CF: carboxyfluorescein
C_max_: maximum plasma concentration
C_max,free_: plasma-free concentration
DMEM: Dulbecco’s modified Eagle’s medium
DMSO: dimethyl sulfoxide
FE_UA_: fractional excretion of uric acid
IC_50_: half maximal inhibition constants
LC/MS/MS: tandem mass spectrometry
OAT: organic anion transporters
PD: pharmacodynamic
PK: pharmacokinetic
PPARγ: peroxisome proliferator-activated receptor gamma
SEM: standard error of the mean
sUA: serum uric acid
XO: xanthine oxidase
XOI: xanthine oxidase inhibitor

## References

[1] Neogi T (2011). Clinical practice: gout. N Engl J Med.

[2] Kuo CF, Grainge MJ, Mallen C, Zhang W, Doherty M (2015). Rising burden of gout in the UK but continuing suboptimal management: a nationwide population study. Ann Rheum Dis.

[3] Lawrence RC, Felson DT, Helmick CG, Arnold LM, Choi H, Deyo RA, Gabriel S, Hirsch R, Hochberg MC, Hunder GG (2008). Estimates of the prevalence of arthritis and other rheumatic conditions in the United States: part II. Arthritis Rheum.

[4] Wallace KL, Riedel AA, Joseph-Ridge N, Wortmann R (2004). Increasing prevalence of gout and hyperuricemia over 10 years among older adults in a managed care population. J Rheumatol.

[5] Zhu Y, Pandya BJ, Choi HK (2011). Prevalence of gout and hyperuricemia in the US general population: the National Health and Nutrition Examination Survey 2007-2008. Arthritis Rheum.

[6] Kuo CF, Grainge MJ, Zhang W, Doherty M (2015). Global epidemiology of gout: prevalence, incidence and risk factors. Nat Rev Rheumatol.

[7] Shields GE, Beard SM (2015). A systematic review of the economic and humanistic burden of gout. Pharmacoeconomics.

[8] Jordan KM, Cameron JS, Snaith M, Zhang W, Doherty M, Seckl J, Hingorani A, Jaques R, Nuki G, British Society for Rheumatology (2007). British Society for Rheumatology and British Health Professionals in Rheumatology guideline for the management of gout. Rheumatology (Oxford).

[9] Khanna D, Khanna PP, Fitzgerald JD, Singh MK, Bae S, Neogi T, Pillinger MH, Merill J, Lee S, Prakash S (2012). 2012 American College of Rheumatology guidelines for management of gout, part 2: therapy and antiinflammatory prophylaxis of acute gouty arthritis. Arthritis Care Res.

[10] Khanna P, Hagerty D, Mischler R, Morlock R (2012). Adherence to EULAR recommendations for the treatment of gout [EULAR abstract FRI0397]. Ann Rheum Dis.

[11] Zhang W, Doherty M, Bardin T, Pascual E, Barskova V, Conaghan P, Gerster J, Jacobs J, Leeb B, Liote F (2006). EULAR evidence based recommendations for gout, part II: management: report of a task force of the EULAR Standing Committee for International Clinical Studies Including Therapeutics (ESCISIT). Ann Rheum Dis.

[12] Zhang W, Doherty M, Pascual E, Bardin T, Barskova V, Conaghan P, Gerster J, Jacobs J, Leeb B, Liote F (2006). EULAR evidence based recommendations for gout, part I: diagnosis: report of a task force of the Standing Committee for International Clinical Studies Including Therapeutics (ESCISIT). Ann Rheum Dis.

[13] Liu S, Perez-Ruiz F, Miner JN (2016). Patients with gout differ from healthy subjects in renal response to changes in serum uric acid. Joint Bone Spine.

[14] Terkeltaub R (2010). Update on gout: new therapeutic strategies and options. Nat Rev Rheumatol.

[15] Choi HK, Mount DB, Reginato AM (2005). Pathogenesis of gout. Ann Intern Med.

[16] Dehghan A, Köttgen A, Yang Q, Hwang SJ, Kao WL, Rivadeneira F, Boerwinkle E, Levy D, Hofman A, Astor BC (2008). Association of three genetic loci with uric acid concentration and risk of gout: a genome-wide association study. Lancet.

[17] Kolz M, Johnson T, Sanna S, Teumer A, Vitart V, Perola M, Mangino M, Albrecht E, Wallace C, Farrall M (2009). Meta-analysis of 28,141 individuals identifies common variants within five new loci that influence uric acid concentrations. PLoS Genet.

[18] Kottgen A, Albrecht E, Teumer A, Vitart V, Krumsiek J, Hundertmark C, Pistis G, Ruggiero D, O'Seaghdha CM, Haller T (2013). Genome-wide association analyses identify 18 new loci associated with serum urate concentrations. Nat Genet.

[19] Enomoto A, Kimura H, Chairoungdua A, Shigeta Y, Jutabha P, Cha SH, Hosoyamada M, Takeda M, Sekine T, Igarashi T (2002). Molecular identification of a renal urate anion exchanger that regulates blood urate levels. Nature.

[20] Ichida K, Hosoyamada M, Hisatome I, Enomoto A, Hikita M, Endou H, Hosoya T (2004). Clinical and molecular analysis of patients with renal hypouricemia in Japan-influence of URAT1 gene on urinary urate excretion. J Am Soc Nephrol.

[21] Anzai N, Ichida K, Jutabha P, Kimura T, Babu E, Jin CJ, Srivastava S, Kitamura K, Hisatome I, Endou H (2008). Plasma urate level is directly regulated by a voltage-driven urate efflux transporter URATv1 (SLC2A9) in humans. J Biol Chem.

[22] Matsuo H, Chiba T, Nagamori S, Nakayama A, Domoto H, Phetdee K, Wiriyasermkul P, Kikuchi Y, Oda T, Nishiyama J (2008). Mutations in glucose transporter 9 gene SLC2A9 cause renal hypouricemia. Am J Hum Genet.

[23] Vitart V, Rudan I, Hayward C, Gray NK, Floyd J, Palmer CN, Knott SA, Kolcic I, Polasek O, Graessler J (2008). SLC2A9 is a newly identified urate transporter influencing serum urate concentration, urate excretion and gout. Nat Genet.

[24] Flynn TJ, Phipps-Green A, Hollis-Moffatt JE, Merriman ME, Topless R, Montgomery G, Chapman B, Stamp LK, Dalbeth N, Merriman TR (2013). Association analysis of the SLC22A11 (organic anion transporter 4) and SLC22A12 (urate transporter 1) urate transporter locus with gout in New Zealand case-control sample sets reveals multiple ancestral-specific effects. Arthritis Res Ther.

[25] Reyes AJ (2003). Cardiovascular drugs and serum uric acid. Cardiovasc Drugs Ther.

[26] Hagos Y, Bahn A, Vormfelde SV, Brockmoller J, Burckhardt G (2007). Torasemide transport by organic anion transporters contributes to hyperuricemia. J Am Soc Nephrol.

[27] Hagos Y, Stein D, Ugele B, Burckhardt G, Bahn A (2007). Human renal organic anion transporter 4 operates as an asymmetric urate transporter. J Am Soc Nephrol.

[28] McAdams-DeMarco MA, Maynard JW, Baer AN, Kao LW, Kottgen A, Coresh J (2013). A urate gene-by-diuretic interaction and gout risk in participants with hypertension: results from the ARIC study. Ann Rheum Dis.

[29] Cunningham RF, Israili ZH, Dayton PG (1981). Clinical pharmacokinetics of probenecid. Clin Pharmacokinet.

[30] Burckhardt G (2012). Drug transport by organic anion transporters (OATs). Pharmacol Ther.

[31] Lee M-HH, Graham GG, Williams KM, Day RO (2008). A benefit-risk assessment of benzbromarone in the treatment of gout: was its withdrawal from the market in the best interest of patients?. Drug Saf.

[32] Derosa G, Maffioli P, Sahebkar A (2015). Plasma uric acid concentrations are reduced by fenofibrate: A systematic review and meta-analysis of randomized placebo-controlled trials. Pharmacol Res.

[33] Wolff ML, Cruz JL, Vanderman AJ, Brown JN (2015). The effect of angiotensin II receptor blockers on hyperuricemia. Ther Adv Chronic Dis.

[34] Fleischmann R, Kerr B, Yeh LT, Suster M, Shen Z, Polvent E, Hingorani V, Quart B, Manhard K, Miner JN (2014). Pharmacodynamic, pharmacokinetic and tolerability evaluation of concomitant administration of lesinurad and febuxostat in gout patients with hyperuricaemia. Rheumatology (Oxford).

[35] Shen Z, Rowlings C, Kerr B, Hingorani V, Manhard K, Quart B, Yeh LT, Storgard C (2015). Pharmacokinetics, pharmacodynamics, and safety of lesinurad, a selective uric acid reabsorption inhibitor, in healthy adult males. Drug Des Dev Ther.

[36] Mount DB (2013). The kidney in hyperuricemia and gout. Curr Opin Nephrol Hypertens.

[37] Perez-Ruiz F, Sundy JS, Miner JN, Cravets M, Storgard C, Group RS (2016). Lesinurad in combination with allopurinol: results of a phase 2, randomised, double-blind study in patients with gout with an inadequate response to allopurinol. Ann Rheum Dis.

[38] Hoy SM (2016). Lesinurad. First Global Approval. Drugs.

[39] Fenner KS, Troutman MD, Kempshall S, Cook JA, Ware JA, Smith DA, Lee CA (2009). Drug-drug interactions mediated through P-glycoprotein: clinical relevance and in vitro-in vivo correlation using digoxin as a probe drug. Clin Pharmacol Ther.

[40] Woodward OM, Kottgen A, Kottgen M (2011). ABCG transporters and disease. FEBS J.

[41] Shen Z, Yeh LT, Wallach K, Zhu N, Kerr B, Gillen M (2016). In vitro and in vivo interaction studies between lesinurad, a selective urate reabsorption inhibitor, and major liver or kidney transporters. Clin Drug Investig.

[42] Kaufmann P, Torok M, Hanni A, Roberts P, Gasser R, Krahenbuhl S (2005). Mechanisms of benzarone and benzbromarone-induced hepatic toxicity. Hepatology.

[43] Broekhuysen J, Pacco M, Sion R, Demeulenaere L, Van Hee M (1972). Metabolism of benzbromarone in man. Eur J Clin Pharmacol.

[44] Karalliedde J, Buckingham RE (2007). Thiazolidinediones and their fluid-related adverse effects: facts, fiction and putative management strategies. Drug Saf.

[45] Kunishima C, Inoue I, Oikawa T, Nakajima H, Komoda T, Katayama S (2007). Activating effect of benzbromarone, a uricosuric drug, on peroxisome proliferator-activated receptors. PPAR Res.

[46] Bardin T, Keenan R, Khanna P, Kopicko J, Fung M, Bhakta N, Adler S, Storgard C, Baumgartner S, So A. Lesinurad, a selective uric acid reabsorption inhibitor, in combination with allopurinol: results from a phase III study in gout patients having an inadequate response to standard of care (CLEAR 2). Poster presented at 16th EULAR Annual European Congress of Rheumatology, Rome, Italy, June 10-13, 2015; Poster FRI0333.

[47] Saag KG, Fitz-Patrick D, Kopicko J, Fung M, Bhakta N, Adler S, Storgard C, Baumgartner S, Becker M. Lesinurad, a selective uric acid reabsorption inhibitor, in combination with allopurinol: results from a phase III study in gout patients having an inadequate response to standard of care (CLEAR 1). Poster presented at 16th EULAR Annual European Congress of Rheumatology, Rome, Italy, June 10-13, 2015; Poster FRI0320.

[48] Heel RC, Brogden RN, Speight TM, Avery GS (1977). Benzbromarone: a review of its pharmacological properties and therapeutic use in gout and hyperuricaemia. Drugs.

[49] Iwanaga T, Kobayashi D, Hirayama M, Maeda T, Tamai I (2005). Involvement of uric acid transporter in increased renal clearance of the xanthine oxidase inhibitor oxypurinol induced by a uricosuric agent, benzbromarone. Drug Metab Dispos.

[50] U.S. Department of Health and Human Services. Guidance for Industry. Drug Interaction Studies-—Study Design, Data Analysis, Implications for Dosing and Labeling Recommendations. US Food and Drug Administration. February 2012. http://www.fda.gov/downloads/drugs/guidancecomplianceregulatoryinformation/guidances/ucm292362.pdf.

[51] Honari J, Blair AD, Cutler RE (1977). Effects of probenecid on furosemide kinetics and natriuresis in man. Clin Pharmacol Ther.

[52] Vallon V, Rieg T, Ahn SY, Wu W, Eraly SA, Nigam SK (2008). Overlapping in vitro and in vivo specificities of the organic anion transporters OAT1 and OAT3 for loop and thiazide diuretics. Am J Physiol Renal Physiol.

[53] Lepist EI, Ray AS (2012). Renal drug-drug interactions: what we have learned and where we are going. Expert Opin Drug Metab Toxicol.

[54] Nigam SK, Wu W, Bush KT, Hoenig MP, Blantz RC, Bhatnagar V (2015). Handling of Drugs, metabolites, and uremic toxins by kidney proximal tubule drug transporters. Clin J Am Soc Nephrol.

[55] Caulfield MJ, Munroe PB, O'Neill D, Witkowska K, Charchar FJ, Doblado M, Evans S, Eyheramendy S, Onipinla A, Howard P (2008). SLC2A9 is a high-capacity urate transporter in humans. PLoS Med.

