# Erratum to: Lesinurad, a novel, oral compound for gout, acts to decrease serum uric acid through inhibition of urate transporters in the kidney

**DOI:** 10.1186/s13075-016-1150-7

**Published:** 2016-10-12

**Authors:** Jeffrey N. Miner, Philip K. Tan, David Hyndman, Sha Liu, Cory Iverson, Payal Nanavati, David T. Hagerty, Kimberly Manhard, Zancong Shen, Jean-Luc Girardet, Li-Tain Yeh, Robert Terkeltaub, Barry Quart

**Affiliations:** 1 Ardea Biosciences, Inc., 9390 Towne Centre Drive, San Diego, CA 92121 USA; 2 University of California San Diego, 9500 Gilman Dr, La Jolla, CA 92093 USA

## Erratum

Unfortunately, after publication of this article [1], it was noticed that the name of Jeffrey N. Miner was captured incorrectly during the production process. The middle initial ‘N’ was missing from the list. The corrected author list can be seen above and the original article has been updated to correct this error.
