# Supplementary material for: Lesinurad, a novel, oral compound for gout, acts to decrease serum uric acid through inhibition of urate transporters in the kidney
Source: Arthritis Res Ther. 2016 Oct 3;18:214. doi: 10.1186/s13075-016-1107-x (PMC5048659; doi:10.1186/s13075-016-1107-x)
Supplement: Additional file 1: — Inhibition of OAT3 by lesinurad. (DOCX 59 kb) [file 13075_2016_1107_MOESM1_ESM.docx]

**Additional file 1**

**Figure S1.** Inhibition of OAT3 by lesinurad. Oocytes injected with RNA encoding OAT3 were either untreated (“0” in a, “no inhibitor in b) or treated with different concentrations of lesinurad or the positive control OAT3 inhibitor bumetanide (b). The background transport from control water-injected oocytes was subtracted to obtain the OAT3-specific transport. Data are from individual experiments, represented at the mean ± SEM.


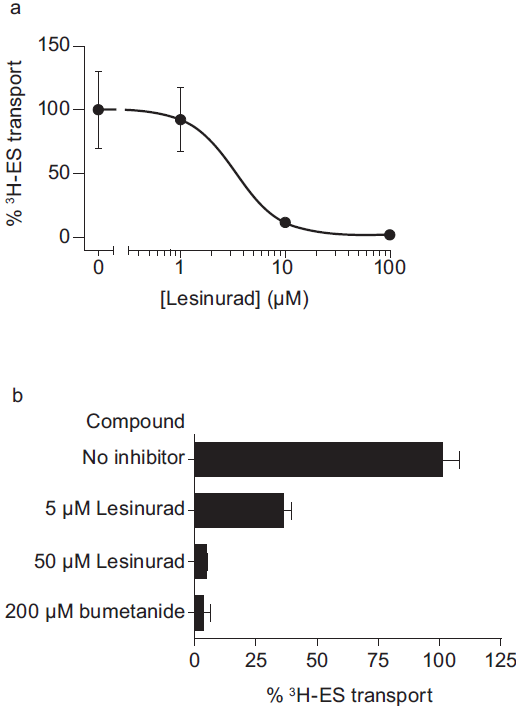


**Additional Data Methods.** Oocytes expressing OAT3 (BD Gentest Transportocytes, catalog number 455663) as well as control water-injected oocytes were prepared according to the manufacturer’s instructions. Oocytes were incubated for 4 to 6 days in ND96 buffer (96 mM NaCl, 2 mM KCl, 1.8 mM CaCl2, 1 mM MgCl2, and 5 mM HEPES, pH 7.4 and 50 µg/mL gentamycin) at 16 °C. For each point, 10 oocytes were transferred to a test tube containing sodium uptake buffer (100 mM NaCl, 2 mM KCl, 1 mM MgCl2, 1 mM CaCl2, and 10 mM HEPES, pH 7.4). Transport was performed at room temperature for 60 minutes using 2 µM 3H-estrone sulfate with or without lesinurad. After transport, the oocytes were washed five times with 5 mL of ice-cold sodium uptake buffer. The oocytes were lysed with 10 % sodium dodecyl sulfate solution prior to scintillation counting.
